# Supplementary material for: Multi-omics reveals the involvement of endophytes in the growth of Moso bamboo (Phyllostachys edulis) shoots
Source: Commun Biol. 2026 Mar 26;9:438. doi: 10.1038/s42003-025-09436-3 (PMC13022179; doi:10.1038/s42003-025-09436-3)
Supplement: Supplementary file 1 — Supplementary Information [file 42003_2025_9436_MOESM1_ESM.pdf]

Supplementary Information

This file contains Supplementary Figures 1-18, Supplementary Table S1-S3 that support the findings of the main manuscript titled “Multi-omics reveals the involvement of endophytes in the growth of Moso bamboo (*Phyllostachys edulis*) shoots.”
Please refer to this document for additional experimental results, data analyses, and methodological details as referenced in the main text.

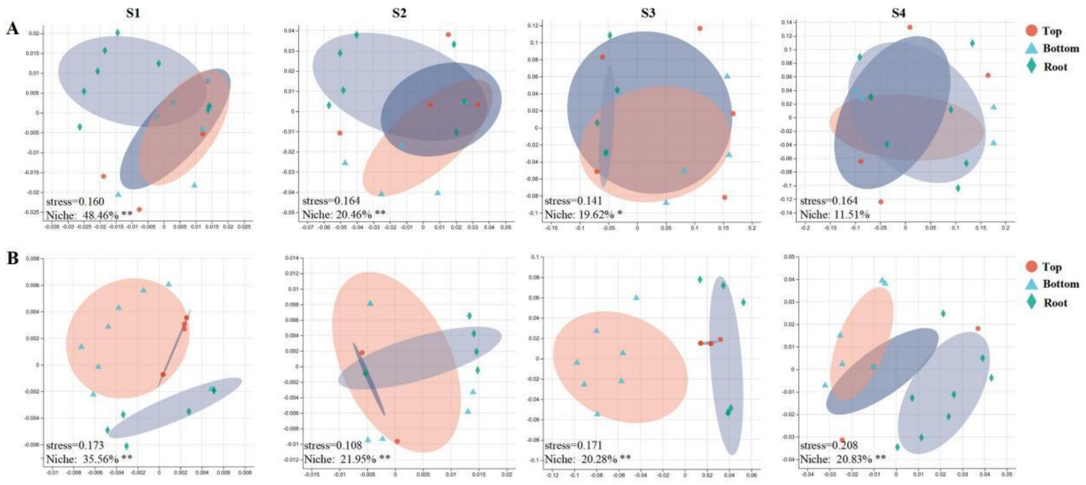

Supplementary Figure 1 | NMDS ordination of bacterial and fungal communities in Moso bamboo shoots across developmental stages.

Non-metric multidimensional scaling (NMDS) plots based on Bray–Curtis dissimilarities illustrate the β-diversity of microbial communities in three compartments (shoot top, shoot bottom, and root) across four developmental stages (S1: dormancy, S2: dormancy-breaking, S3: accelerated growth, S4: rapid growth). Each point represents one sample (n = 8 biologically independent samples per compartment per stage): circles indicate shoot tops, triangles indicate shoot bottoms, and diamonds indicate roots. Colored ellipses show 95% confidence intervals for each compartment. Stress values and PERMANOVA-based niche effects (R^2 and significance levels) are shown in each panel.

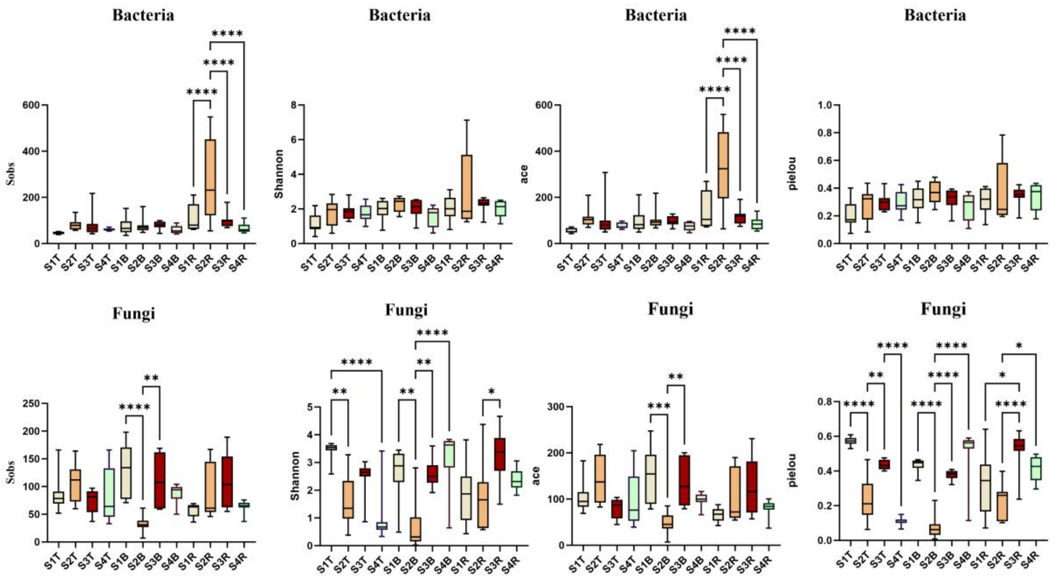

Supplementary Figure 2 | Comparison of multiple α-diversity indices of bacterial and fungal communities in Moso bamboo.

α-diversity indices of bacterial and fungal communities in different compartments (shoot top, shoot bottom, root) and developmental stages (S1–S4). Four indices are shown: observed species (Sobs), Shannon diversity index, ACE richness estimator, and Pielou's evenness index. Boxplots represent the distribution of diversity values across n = 8 biologically independent samples per group. Statistical significance was determined by one-way ANOVA with Tukey's post-hoc test. *$P < 0.05$, **$P < 0.01$, ***$P < 0.001$, ****$P < 0.0001$.

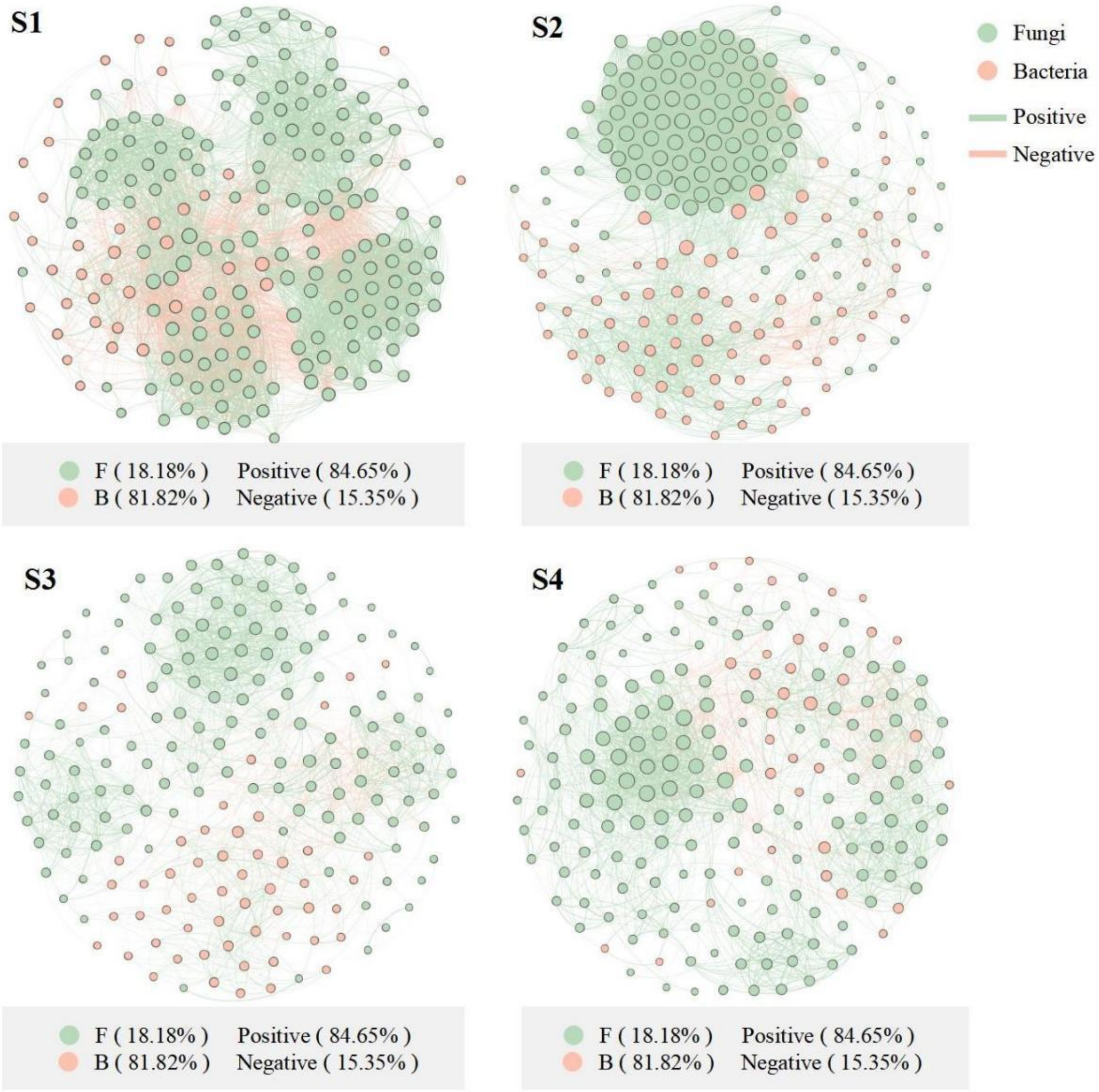

Supplementary Figure 3 | Temporal dynamics of endophytic microbial co-occurrence networks in Moso bamboo roots across four developmental stages.

Microbial co-occurrence networks were constructed based on Spearman correlation coefficients ($\rho > 0.6$ or < -0.6, $P < 0.01$) for each developmental stage: dormancy (S1), dormancy-breaking (S2), accelerated growth (S3), and rapid growth (S4), using n = 8 biologically independent root samples per stage. Each node represents an operational taxonomic unit (OTU), with green and pink nodes indicating fungal and bacterial taxa, respectively. Edges represent significant correlations, with gray for positive and red for negative associations. Summary statistics below each network indicate the proportion of fungal (F) and bacterial (B) nodes, and the relative proportions of positive versus negative interactions.

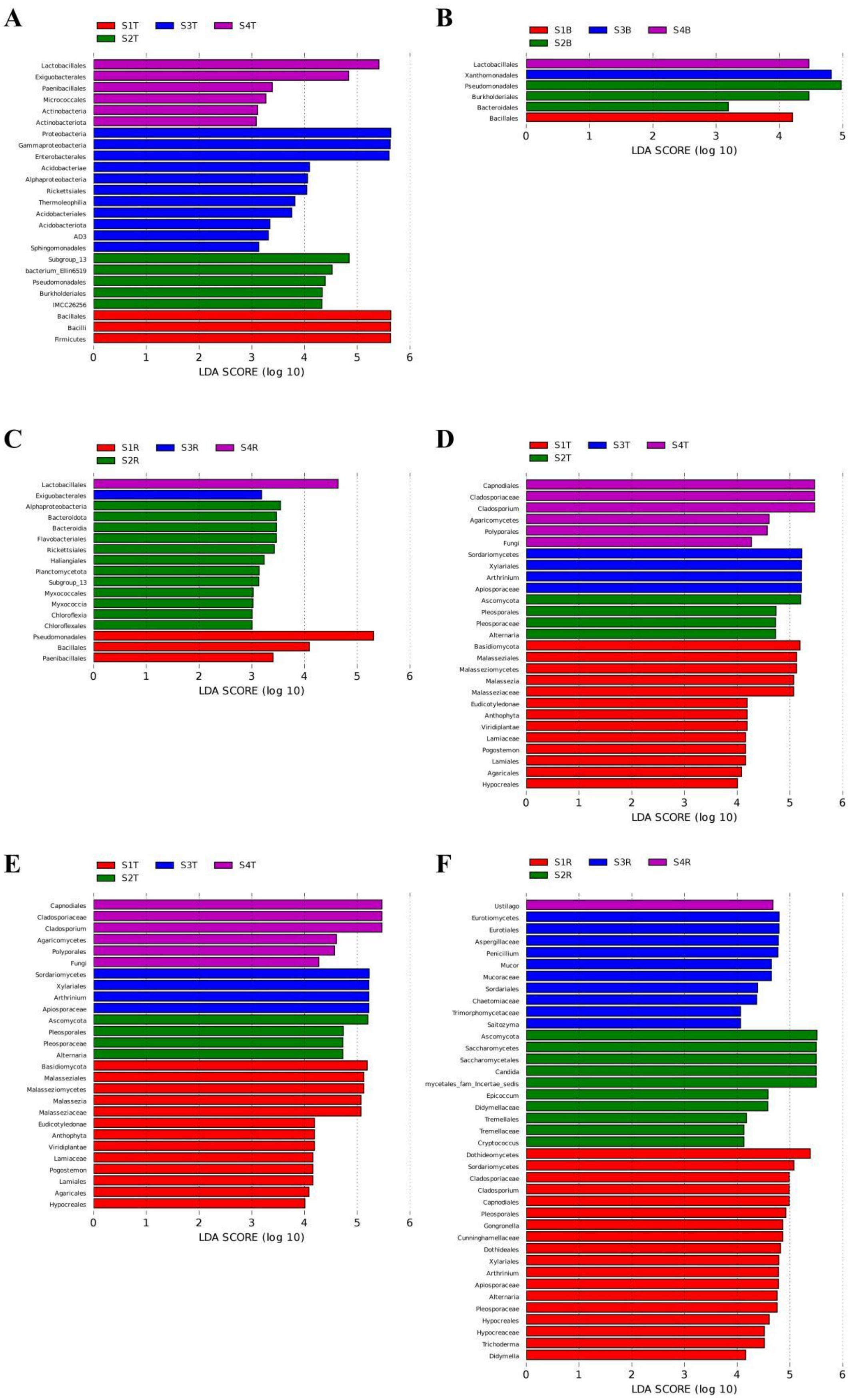
A
S1T
S3T
S4T
S2T
Lactobacillales
Exiguobacterales
Paenibacillales
Micrococcales
Actinobacteria
Actinobacteriota
Proteobacteria
Gammaproteobacteria
Enterobacterales
Acidobacteriae
Alphaproteobacteria
Rickettsiales
Thermoleophilia
Acidobacteriales
Acidobacteriota
AD3
Sphingomonadales
Subgroup_13
bacterium_Ellin6519
Pseudomonadales
Burkholderiales
IMCC26256
Bacillales
Bacilli
Firmicutes
0 1 2 3 4 5 6
LDA SCORE (log 10)
B
S1B
S3B
S4B
S2B
Lactobacillales
Xanthomonadales
Pseudomonadales
Burkholderiales
Bacteroidales
Bacillales
0 1 2 3 4 5
LDA SCORE (log 10)
C
S1R
S3R
S4R
S2R
Lactobacillales
Exiguobacterales
Alphaproteobacteria
Bacteroidota
Bacteroidia
Flavobacteriales
Rickettsiales
Haliangiales
Planctomycetota
Subgroup_13
Myxococcales
Myxococcia
Chloroflexia
Chloroflexales
Pseudomonadales
Bacillales
Paenibacillales
0 1 2 3 4 5 6
LDA SCORE (log 10)
D
S1T
S3T
S4T
S2T
Capnodiales
Cladosporiaceae
Cladosporium
Agaricomycetes
Polyporales
Fungi
Sordariomycetes
Xylariales
Arthrinium
Apiosporaceae
Ascomycota
Pleosporales
Pleosporaceae
Alternaria
Basidiomycota
Malasseziales
Malasseziomycetes
Malassezia
Malasseziaceae
Eudicotyledonae
Anthophyta
Viridiplantae
Lamiaceae
Pogostemon
Lamiales
Agaricales
Hypocreales
0 1 2 3 4 5 6
LDA SCORE (log 10)
E
S1T
S3T
S4T
S2T
Capnodiales
Cladosporiaceae
Cladosporium
Agaricomycetes
Polyporales
Fungi
Sordariomycetes
Xylariales
Arthrinium
Apiosporaceae
Ascomycota
Pleosporales
Pleosporaceae
Alternaria
Basidiomycota
Malasseziales
Malasseziomycetes
Malassezia
Malasseziaceae
Eudicotyledonae
Anthophyta
Viridiplantae
Lamiaceae
Pogostemon
Lamiales
Agaricales
Hypocreales
0 1 2 3 4 5 6
LDA SCORE (log 10)
F
S1R
S3R
S4R
S2R
Ustilago
Eurotiomycetes
Eurotiales
Aspergillaceae
Penicillium
Mucor
Mucoraceae
Sordariales
Chaetomiaceae
Trimorphomycetaceae
Saitozyma
Ascomycota
Saccharomycetes
Saccharomycetales
Candida
mycetales_fam_Incertae_sedis
Epicoccum
Didymellaceae
Tremellales
Tremellaceae
Cryptococcus
Dothideomycetes
Sordariomycetes
Cladosporiaceae
Cladosporium
Capnodiales
Pleosporales
Gongronella
Cunninghamellaceae
Dothideales
Xylariales
Arthrinium
Apiosporaceae
Alternaria
Pleosporaceae
Hypocreales
Hypocreaceae
Trichoderma
Didymella
0 1 2 3 4 5 6
LDA SCORE (log 10)

Supplementary Figure 4 | Discriminative microbial taxa identified by LEfSe across developmental stages and compartments of Moso bamboo shoots.

Linear discriminant analysis (LDA) effect size (LEfSe) was used to identify bacterial (A–C) and fungal (D–F) taxa with significant differences among compartments (shoot top, shoot bottom, and root) and developmental stages (S1–S4). Panels (A–C) show bacterial taxa in the shoot top (A), shoot bottom (B), and root (C), respectively. Panels (D–F) show fungal taxa in the shoot top (D), shoot bottom (E), and root (F), respectively. Each bar represents a taxon with an LDA score > 2.0 and P < 0.05. Bar colors indicate the group in which the taxa were enriched.
interactions.

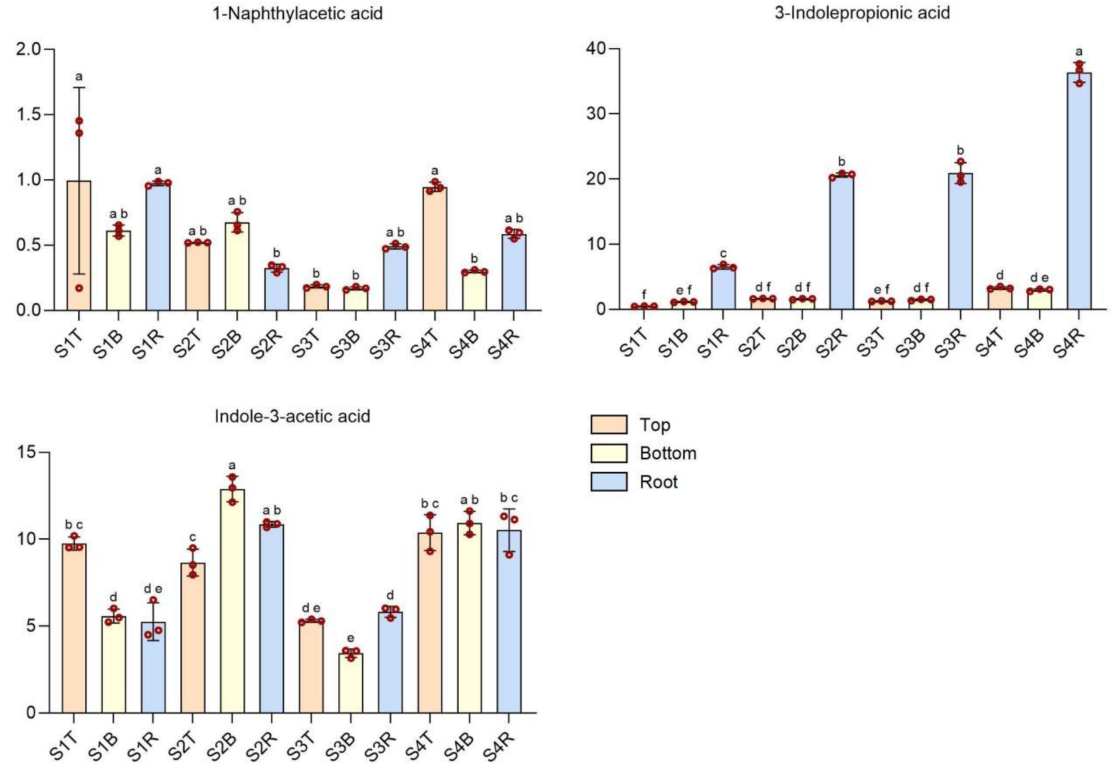

Supplementary Figure 5 | Spatiotemporal dynamics of gibberellin concentrations in different compartments of Moso bamboo shoots.

Quantification of gibberellins in the shoot top, shoot bottom, and root compartments of Moso bamboo across four developmental stages (S1–S4). Each bar represents the mean ± s.e.m. (n = 3 biologically independent samples per compartment per stage). Different letters above the bars indicate statistically significant differences (one-way ANOVA followed by Tukey’s HSD test, $P < 0.05$). Error bars represent the standard error of the mean.

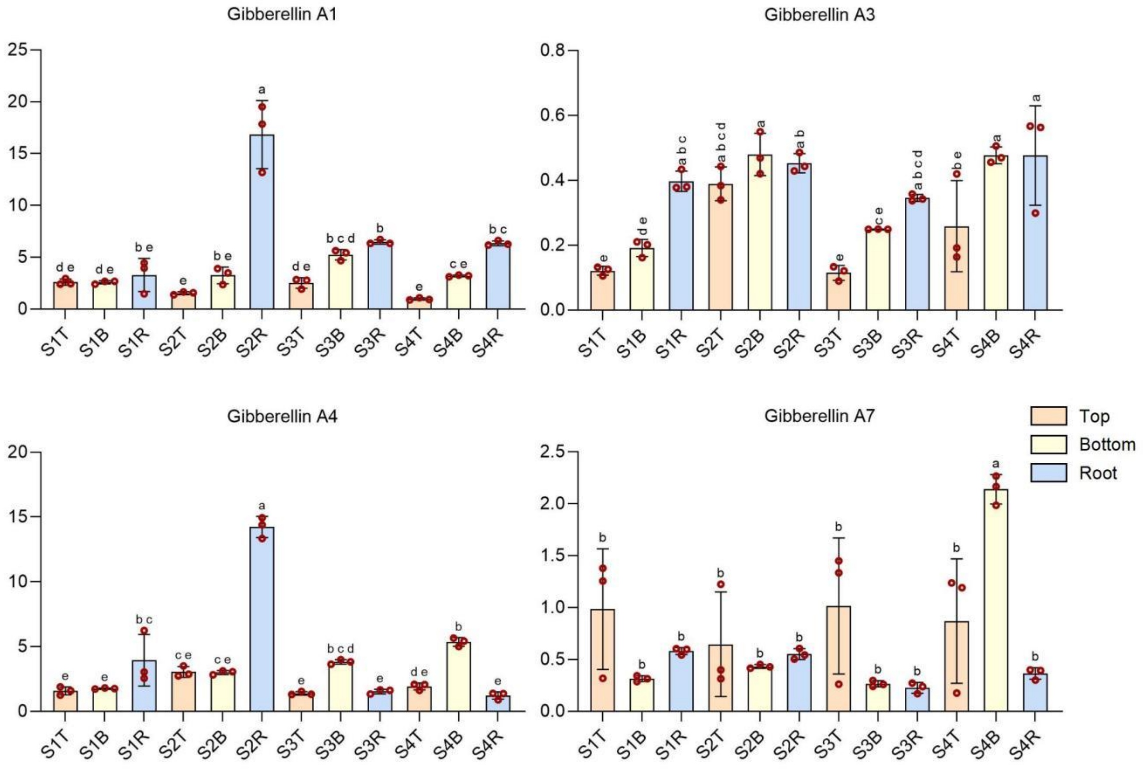

Supplementary Figure 6| Spatiotemporal dynamics of auxin concentrations in different compartments of Moso bamboo shoots.

Quantification of auxins in the shoot top, shoot bottom, and root compartments of Moso bamboo across four developmental stages (S1–S4). Each bar represents mean ± s.e.m. (n = 3 biologically independent samples per compartment per stage). Different letters above bars indicate statistically significant differences ($P < 0.05$) based on one-way ANOVA followed by Tukey's HSD test. Error bars represent the standard error of the mean.

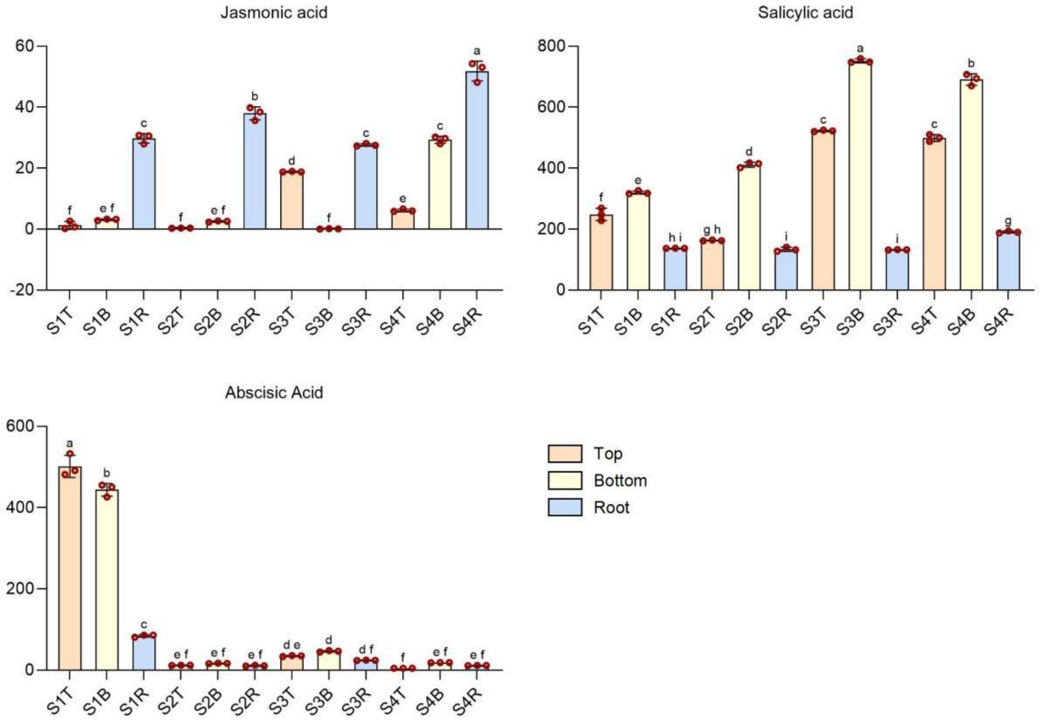

Supplementary Figure 7 | Spatiotemporal dynamics of jasmonic acid (JA), abscisic acid (ABA), and salicylic acid (SA) concentrations in different compartments of Moso bamboo shoots.

Quantification of three phytohormones - jasmonic acid (JA), abscisic acid (ABA), and salicylic acid (SA) - in the shoot top, shoot bottom, and root compartments of Moso bamboo across four developmental stages (S1–S4). Each bar represents mean ± s.e.m. (n = 3 biologically independent samples per compartment per stage). Different letters above bars indicate statistically significant differences ($P < 0.05$) based on one-way ANOVA followed by Tukey's HSD test. Error bars represent the standard error of the mean.

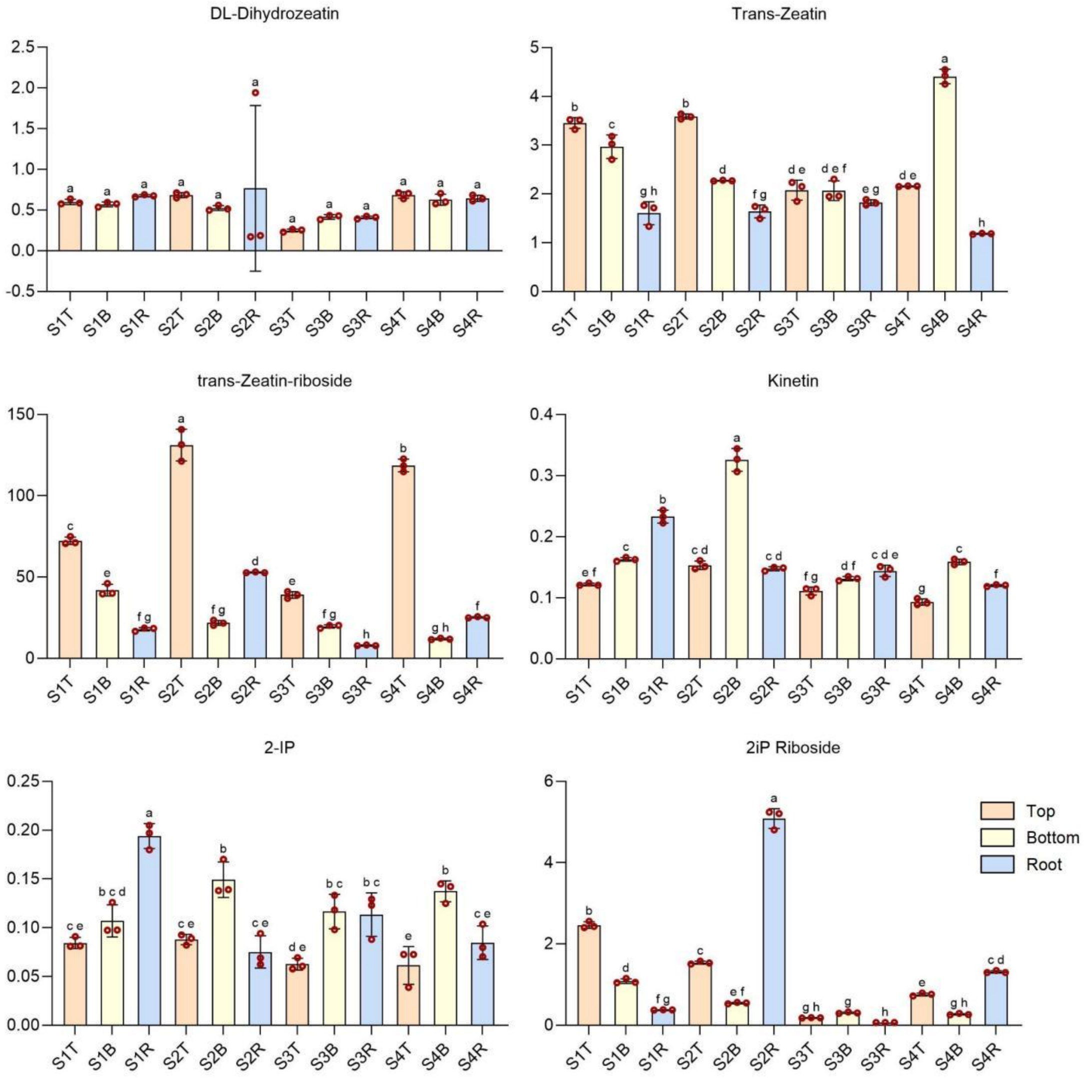

Supplementary Figure 8 | Spatiotemporal dynamics of cytokinin-related phytohormones in different compartments of Moso bamboo shoots.

Quantification of six cytokinin-related phytohormones (DL-dihydrozeatin, trans-zeatin, trans-zeatin riboside, kinetin, 2-isopentenyladenine (2-iP), and 2-isopentenyladenosine (2-iP riboside)) in the shoot top, shoot bottom, and root compartments of Moso bamboo across four developmental stages (S1–S4). Each bar represents mean ± s.e.m. (n = 3 biologically independent samples per compartment per stage). Different letters above bars indicate statistically significant differences ($P < 0.05$) based on one-way ANOVA followed by Tukey's HSD test. Error bars represent the standard error of the mean.

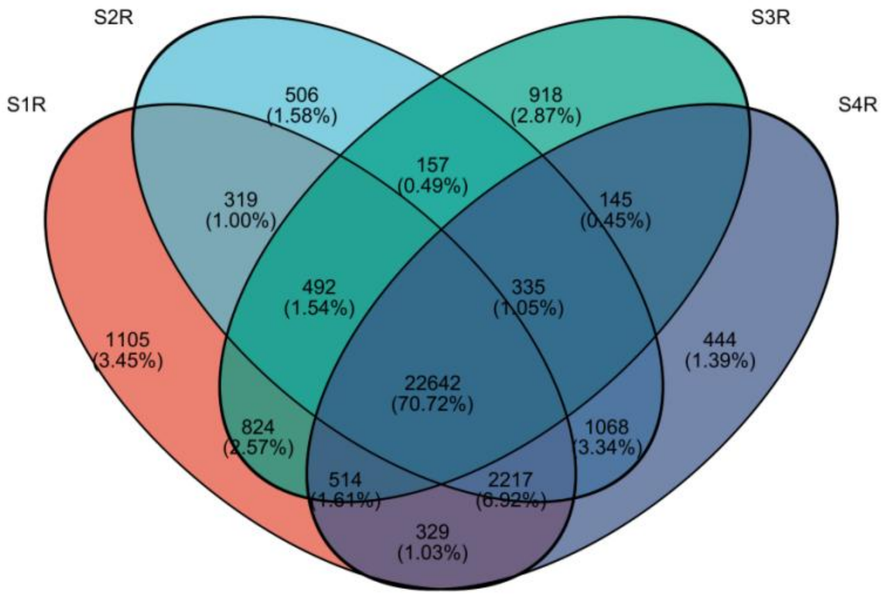

Supplementary Figure 9 | Venn diagram showing overlap of expressed genes in Moso bamboo roots across four developmental stages.

Each circle represents the set of genes expressed in root tissues at a given stage: S1R (dormancy), S2R (dormancy-breaking), S3R (accelerated growth), and S4R (rapid growth). Numbers indicate the counts and percentages of shared or unique genes in each combination. A total of 22,642 genes (70.72%) were commonly expressed across all four stages, while stage-specific expression patterns were also evident, with S1R and S3R exhibiting the highest numbers of unique transcripts.

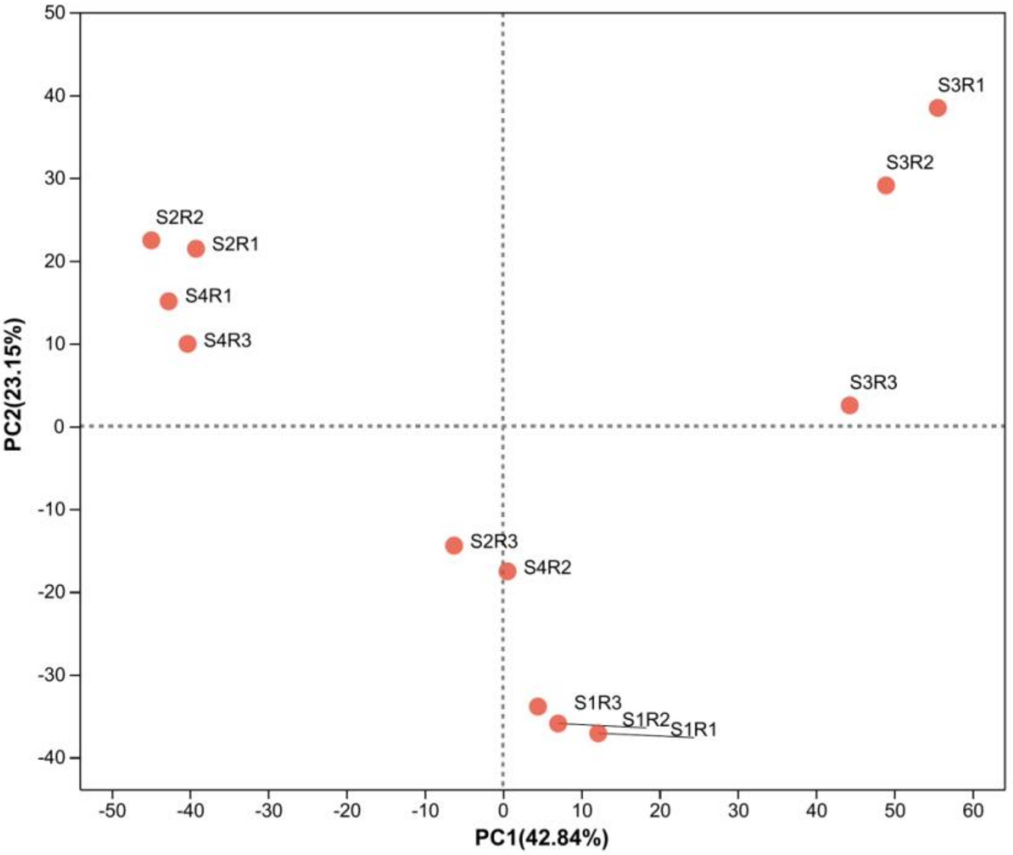

Supplementary Figure 10 | Principal component analysis (PCA) of transcriptomes in Moso bamboo roots at different developmental stages.

Each dot represents a biological replicate (n = 3 biologically independent root samples per stage): S1R (dormancy), S2R (dormancy-breaking), S3R (accelerated growth), and S4R (rapid growth). PCA was performed based on normalized gene expression levels (FPKM). PC1 and PC2 explain 42.84% and 23.15% of the variance, respectively. Samples from different stages form distinct clusters, indicating stage-specific transcriptional programs in the roots during shoot development.

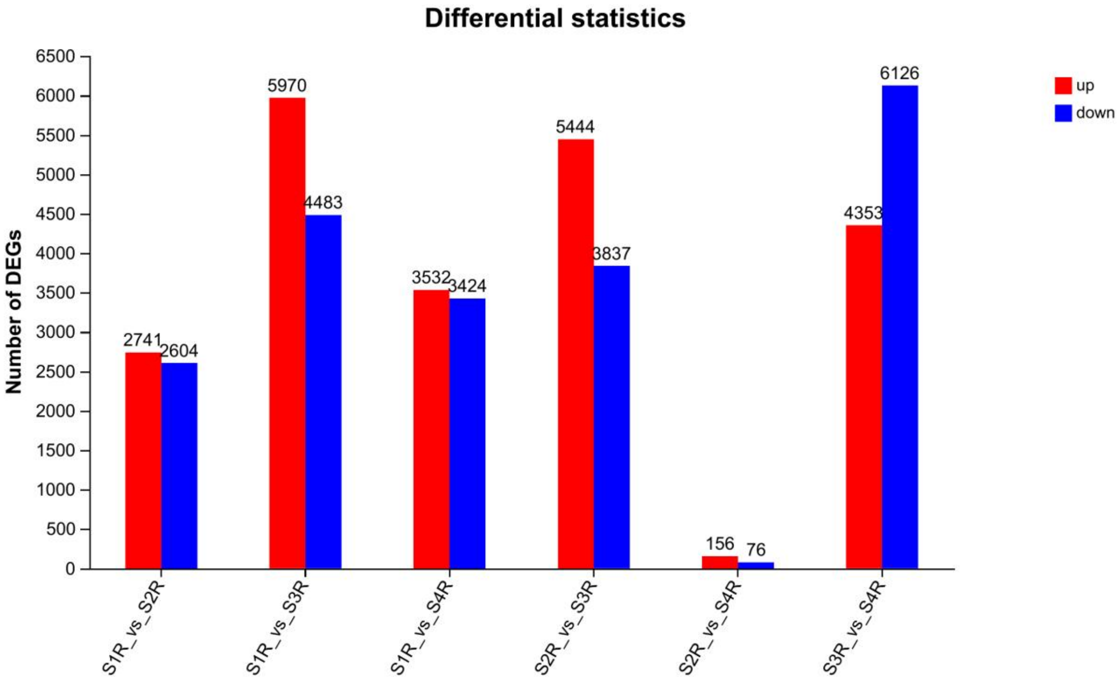

Supplementary Figure 11 | Differential expression analysis of genes in Moso bamboo roots across developmental stages.

Bar plots showing the number of differentially expressed genes (DEGs) between developmental stages of Moso bamboo roots. Comparisons include S1R vs. S2R, S1R vs. S3R, S1R vs. S4R, S2R vs. S3R, S2R vs. S4R, and S3R vs. S4R. Red and blue bars represent upregulated and downregulated genes, respectively. The highest number of DEGs was observed in the S1R vs. S3R comparison, indicating major transcriptional shifts during the transition from dormancy to accelerated growth.

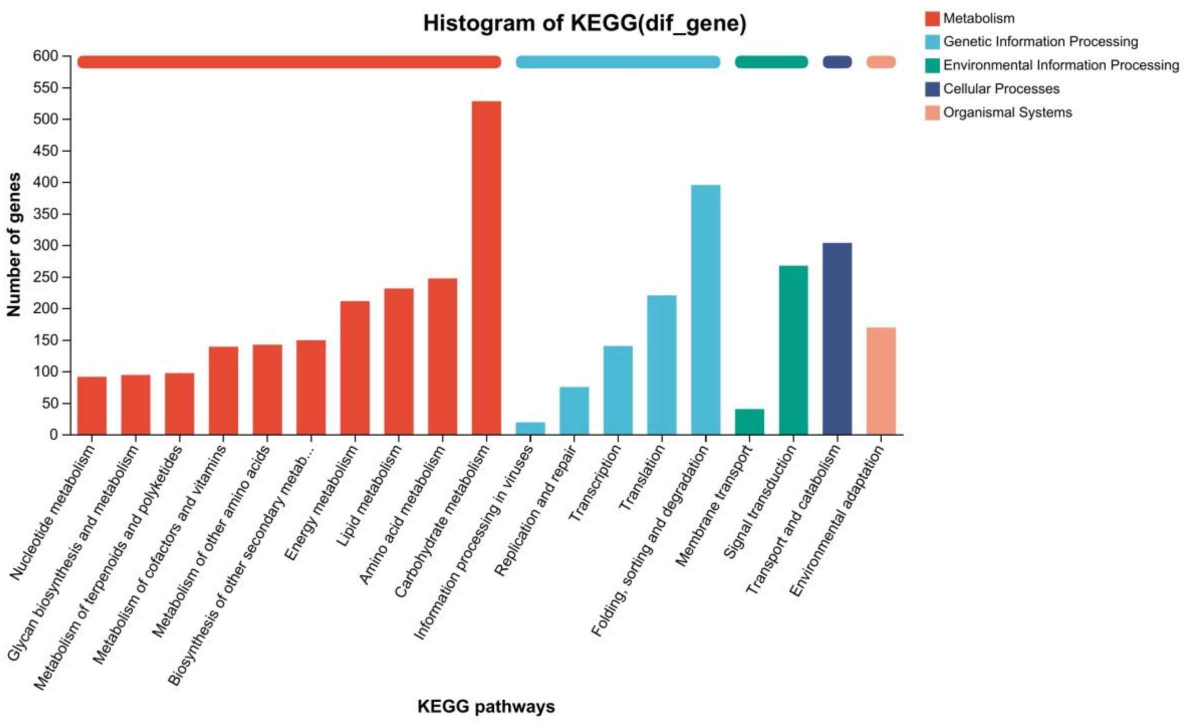

Supplementary Figure 12 | KEGG pathway enrichment analysis of differentially expressed genes in Moso bamboo roots.

Bar plot showing the number of differentially expressed genes (DEGs) enriched in Kyoto Encyclopedia of Genes and Genomes (KEGG) pathways. DEGs were primarily enriched in pathways related to metabolism (red), followed by genetic information processing (cyan), environmental information processing (green), cellular processes (blue), and organismal systems (orange). The most enriched categories included “carbohydrate metabolism,” “amino acid metabolism,” “folding, sorting and degradation,” and “signal transduction,” suggesting active molecular processes underlying root development during bamboo shoot growth.

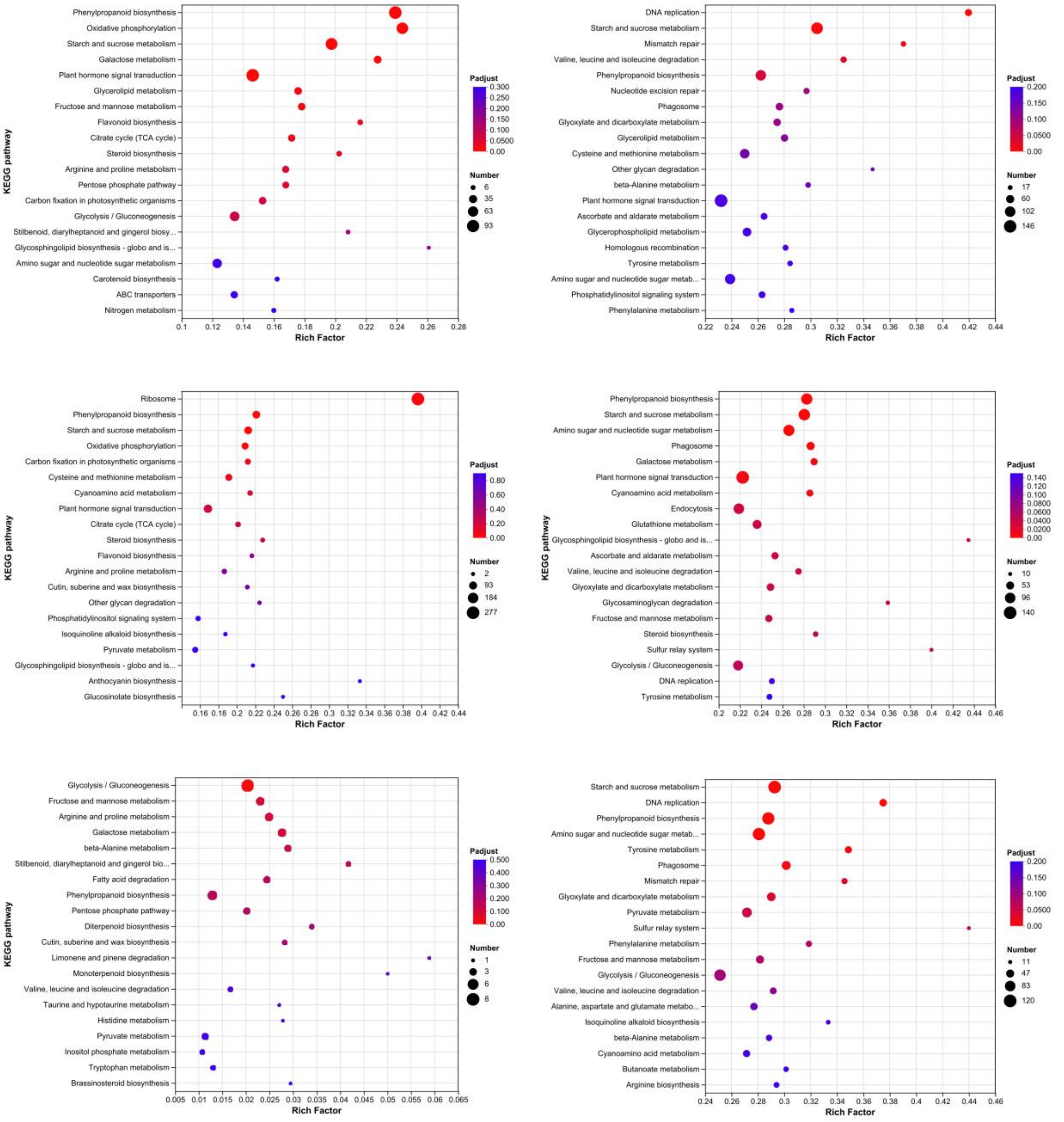

Supplementary Figure 13 | KEGG enrichment analysis of differentially expressed genes (DEGs) in Moso bamboo roots across developmental stages.

Bubble plots show KEGG pathway enrichment of DEGs identified in six pairwise comparisons: (A) S1R vs. S2R, (B) S1R vs. S3R, (C) S1R vs. S4R, (D) S2R vs. S3R, (E) S2R vs. S4R, and (F) S3R vs. S4R. The x-axis represents the Rich Factor (ratio of DEGs to total genes in a given pathway), and the y-axis shows the KEGG pathway names. Bubble size indicates the number of DEGs enriched in each pathway, while the color gradient reflects the adjusted P value (Padj), with red indicating higher significance.

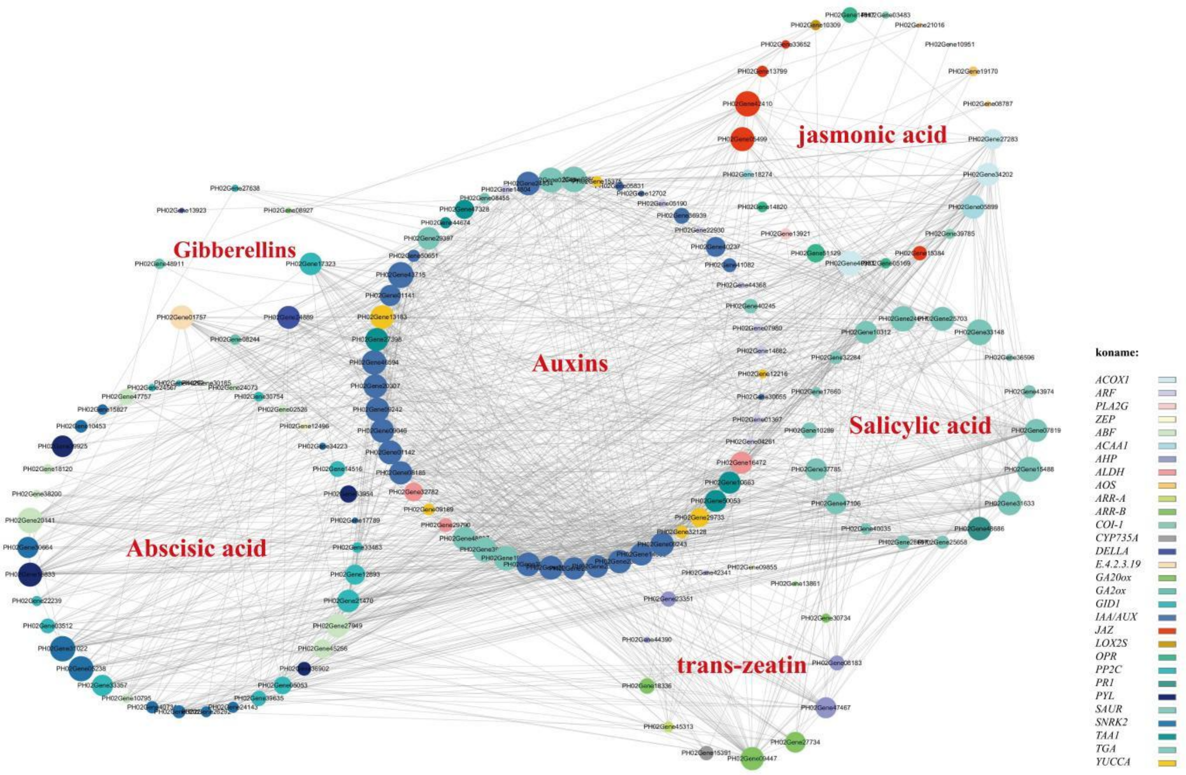

Supplementary Figure 14 | Gene co-expression network of hormone-related genes in Moso bamboo roots.

Network analysis showing co-expression relationships among differentially expressed genes (DEGs) involved in phytohormone biosynthesis and signaling pathways. Genes are clustered into major hormone categories: auxins, gibberellins, abscisic acid, jasmonic acid, salicylic acid, and trans-zeatin. Each node represents a hormone-related gene, colored according to its corresponding gene family (legend at right). Edges represent significant co-expression correlations (Pearson's $|r| > 0.8$, $P < 0.01$). Auxin-related genes form the most extensive and highly connected network cluster, indicating a central role in hormonal regulation during root development.

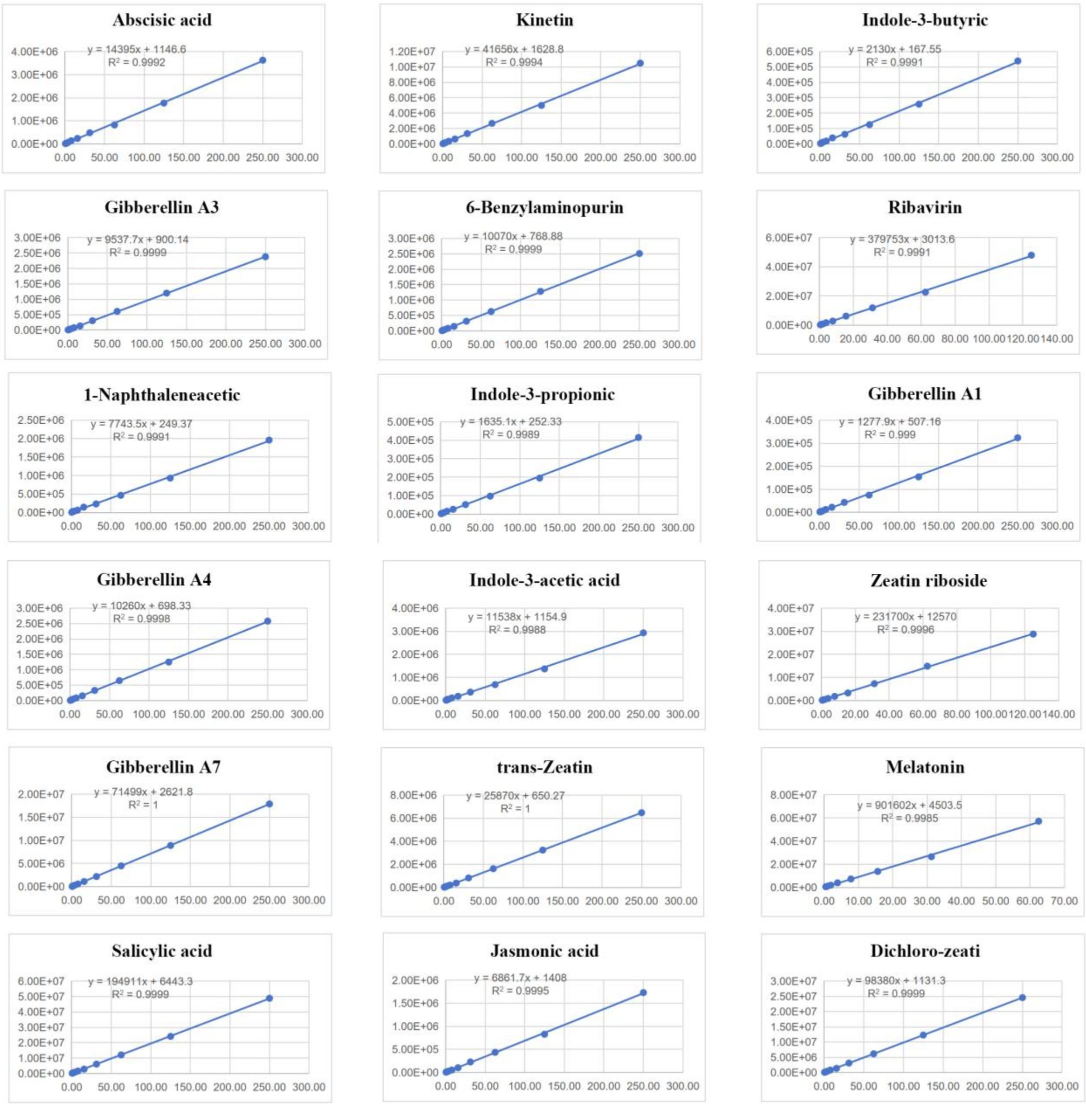

Supplementary Figure 15 | Calibration curves of targeted phytohormones quantified by LC–MS/MS.

Authentic standards of representative hormones (e.g., IAA, ABA, GA, SA, JA, cytokinins) were analyzed across a concentration range of 0.1–100 ng/mL. Each curve shows the regression equation and coefficient of determination (R^2), all exceeding 0.99, indicating excellent linearity. These calibration curves were used for quantification of hormone concentrations in Moso bamboo tissues.

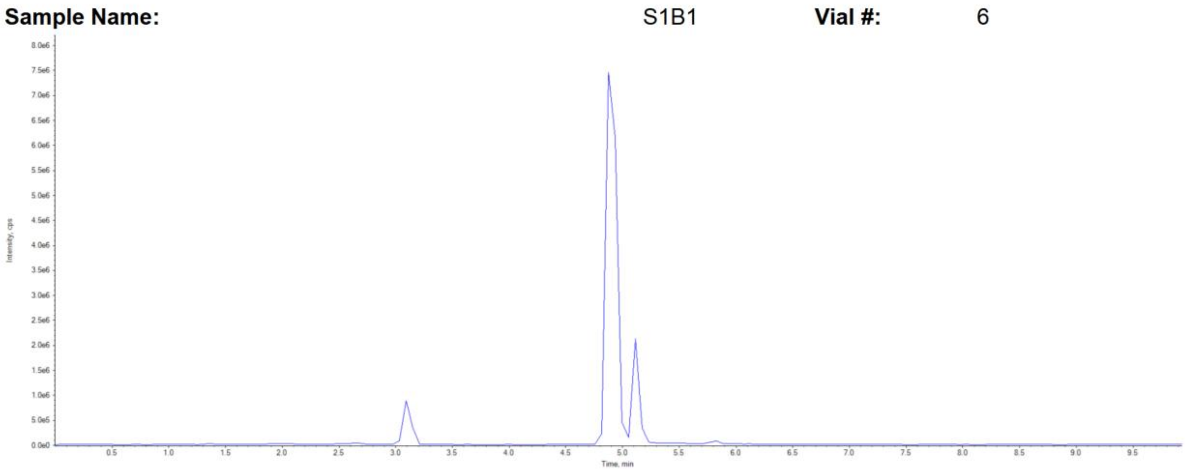

Supplementary Figure 16 | Representative LC–MS/MS chromatogram of phytohormone detection in Moso bamboo roots (sample ID: S1B1).

The chromatogram shows retention times and signal intensities of multiple hormone peaks, confirming successful separation and quantification. Stable isotope-labeled internal standards were spiked into all samples to ensure accurate peak identification and correction for matrix effects.

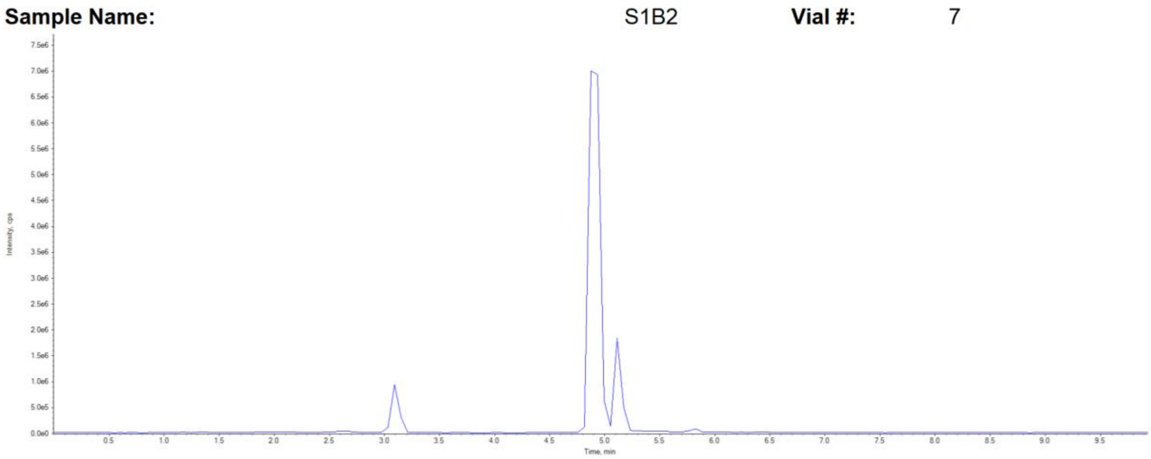

Supplementary Figure 17 | Representative LC–MS/MS chromatogram of phytohormone detection in Moso bamboo roots (sample ID: S1B2).

The chromatogram shows retention times and signal intensities of multiple hormone peaks, confirming successful separation and quantification. Stable isotope-labeled internal standards were spiked into all samples to ensure accurate peak identification and correction for matrix effects.

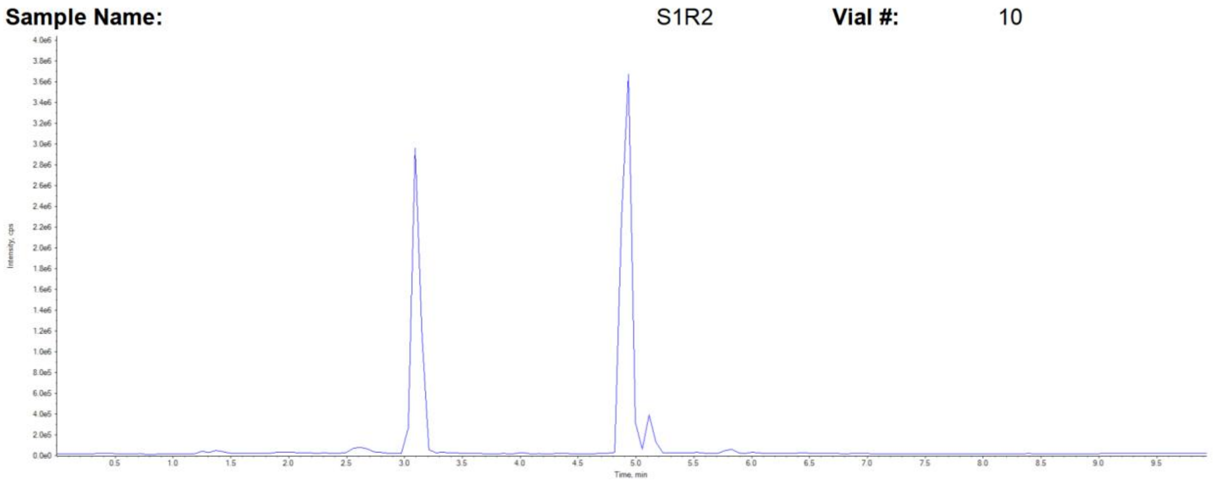

Supplementary Figure 18 | Representative LC–MS/MS chromatogram of phytohormone detection in Moso bamboo roots (sample ID: S1R2).

The chromatogram shows retention times and signal intensities of multiple hormone peaks, confirming successful separation and quantification. Stable isotope-labeled internal standards were spiked into all samples to ensure accurate peak identification and correction for matrix effects.

Table S1 Topological properties of microbial co-occurrence networks across bamboo shoot compartments and developmental stages

		Nodes	Edges	Average degree	Average Weighted Degree	Density	Modularity	Average Clustering Coefficient	Average Path length
T	S1	200	4877	48.77	43.26	0.245	0.438	0.813	2.096
	S2	49	159	6.46	5.191	0.135	0.479	0.505	2.732
	S3	41	63	3.073	2.503	0.077	0.615	0.529	4.018
	S4	48	112	4.667	3.78	0.099	0.618	0.537	4.082
B	S1	48	153	6.375	5.273	0.136	0.535	0.638	3.508
	S2	48	153	6.375	5.273	0.136	0.603	0.68	3.775
	S3	48	125	5.208	4.139	0.111	0.477	0.481	3.537
	S4	48	166	6.917	5.697	0.147	0.501	0.62	2.562
R	S1	46	154	6.696	5.475	0.149	0.425	0.586	2.873
	S2	50	349	13.96	12.171	0.285	0.21	0.723	2.528
	S3	43	162	7.535	6.078	0.176	0.44	0.562	2.516
	S4	49	229	9.347	7.45	0.195	0.388	0.561	2.457

Table S2 Topological properties of the top 15 highest-degree hub nodes in the hormone-related gene co-expression network shown in Supplementary Fig. 14.

Nodes	KO	Degree	Average Shortest Path Length	Betweenness Centrality	Closeness Centrality	Clustering Coefficient
PH02Gene23240	K14484	53	2.234	0.0401	0.447	0.483
PH02Gene14060	K14484	52	2.295	0.026	0.436	0.494
PH02Gene09447	K14491	47	2.308	0.029	0.433	0.527
PH02Gene09243	K14484	46	2.342	0.017	0.427	0.551
PH02Gene07819	K14431	45	2.288	0.035	0.437	0.510
PH02Gene13183	K11816	43	2.362	0.024	0.423	0.575
PH02Gene23239	K14484	42	2.395	0.013	0.417	0.549
PH02Gene08185	K14484	42	2.376	0.029	0.421	0.546
PH02Gene34202	K00232	41	2.517	0.016	0.397	0.574
PH02Gene38761	K14484	40	2.383	0.013	0.420	0.579
PH02Gene26194	K14484	39	2.497	0.036	0.401	0.582
PH02Gene40931	K00232	39	2.517	0.010	0.397	0.629

Table S3 Topological properties of the top 15 highest-degree nodes in the integrated plant-microbe-hormone interaction network shown in Fig. 6.

Nodes	Module	Degree	Average Shortest Path Length	Betweenness Centrality	Closeness Centrality	Clustering Coefficient
Trans Zeatin Riboside	Hormone	59	2.006	0.118	0.499	0.112
Indole 3 aceticacid	Hormone	56	2.103	0.158	0.476	0.017
Gibberellin A1	Hormone	48	2.177	0.059	0.459	0.253
Gibberellin A4	Hormone	47	2.246	0.053	0.445	0.190
Cryptococcus	Fungi	44	2.183	0.053	0.458	0.130
Ralstonia	Bacteria	41	2.269	0.024	0.441	0.093
DL Dihydrozeatin	Hormone	41	2.366	0.162	0.423	0.122
Allorhizobium	Bacteria	39	2.280	0.020	0.439	0.099
Gibberellin A3	Hormone	39	2.371	0.111	0.422	0.001
Arthrobacter	Bacteria	35	2.394	0.012	0.418	0.074
Paenibacillus	Bacteria	34	2.354	0.011	0.425	0.121
Lysinibacillus	Bacteria	33	2.537	0.011	0.394	0.044
