# Supplementary material for: Multi-omics reveals the involvement of endophytes in the growth of Moso bamboo (Phyllostachys edulis) shoots
Source: Commun Biol. 2026 Mar 26;9:438. doi: 10.1038/s42003-025-09436-3 (PMC13022179; doi:10.1038/s42003-025-09436-3)
Supplement: Supplementary file 2 — Description of Additional Supplementary Files [file 42003_2025_9436_MOESM2_ESM.docx]

Description of Additional Supplementary Files

**File name:** Supplementary Data 1

**Description:** This file contains all numerical source data underlying the graphs and charts presented in the main figures, consolidated into separate tabs. It includes bacterial and fungal OTU abundance tables, alpha-diversity indices, hormone concentration measurements, gene expression values (FPKM), KEGG functional annotations, and node attribute data for the co- expression/interaction network in Figure 7.
