# Supplementary material for: Multi-omics reveals the involvement of endophytes in the growth of Moso bamboo (Phyllostachys edulis) shoots
Source: Commun Biol. 2026 Mar 26;9:438. doi: 10.1038/s42003-025-09436-3 (PMC13022179; doi:10.1038/s42003-025-09436-3)
Supplement: Supplementary file 4 — Reporting Summary [file 42003_2025_9436_MOESM4_ESM.pdf]

nature portfolio

Corresponding author(s): Xuhua Du, Xingcui Ding

Last updated by author(s): Dec 15, 2025

Reporting Summary

Nature Portfolio wishes to improve the reproducibility of the work that we publish. This form provides structure for consistency and transparency in reporting. For further information on Nature Portfolio policies, see our Editorial Policies and the Editorial Policy Checklist.

Statistics

For all statistical analyses, confirm that the following items are present in the figure legend, table legend, main text, or Methods section.

n/a	Confirmed	
☐	☒	The exact sample size (*n*) for each experimental group/condition, given as a discrete number and unit of measurement
☐	☒	A statement on whether measurements were taken from distinct samples or whether the same sample was measured repeatedly
☐	☒	The statistical test(s) used AND whether they are one- or two-sided<br>*Only common tests should be described solely by name; describe more complex techniques in the Methods section.*
☐	☒	A description of all covariates tested
☐	☒	A description of any assumptions or corrections, such as tests of normality and adjustment for multiple comparisons
☐	☒	A full description of the statistical parameters including central tendency (e.g. means) or other basic estimates (e.g. regression coefficient) AND variation (e.g. standard deviation) or associated estimates of uncertainty (e.g. confidence intervals)
☐	☒	For null hypothesis testing, the test statistic (e.g. *F*, *t*, *r*) with confidence intervals, effect sizes, degrees of freedom and *P* value noted<br>*Give P values as exact values whenever suitable.*
☐	☒	For Bayesian analysis, information on the choice of priors and Markov chain Monte Carlo settings
☐	☒	For hierarchical and complex designs, identification of the appropriate level for tests and full reporting of outcomes
☐	☒	Estimates of effect sizes (e.g. Cohen's *d*, Pearson's *r*), indicating how they were calculated

Our web collection on statistics for biologists contains articles on many of the points above.

Software and code

Policy information about availability of computer code

Data collection	Amplicon sequencing, transcriptomic sequencing, and LC–MS/MS data were generated using Illumina sequencing platforms and Agilent mass spectrometry systems following standard protocols.
Data analysis	All analyses were performed using established and publicly available pipelines. Amplicon data were processed with QIIME2 (v2022.2). Statistical analysis and visualization were conducted in R (v4.2.2) with publicly available packages (including vegan, DESeq2, ggplot2, and others). Transcriptomic data were analyzed using HISAT2 (v2.2.1) for read alignment and DESeq2 for differential expression analysis. No custom or unpublished code was developed in this study.

For manuscripts utilizing custom algorithms or software that are central to the research but not yet described in published literature, software must be made available to editors and reviewers. We strongly encourage code deposition in a community repository (e.g. GitHub). See the Nature Portfolio guidelines for submitting code & software for further information.

Data

Policy information about availability of data

All manuscripts must include a data availability statement. This statement should provide the following information, where applicable:

- Accession codes, unique identifiers, or web links for publicly available datasets
- A description of any restrictions on data availability
- For clinical datasets or third party data, please ensure that the statement adheres to our policy

All amplicon sequencing and transcriptomic data generated in this study have been deposited in the NCBI Sequence Read Archive (SRA) under the BioProject accession PRJNA1168528, PRJNA1168663, and PRJNA1168675.All other data supporting the findings of this study are available within the paper and its Supplementary Information files. Source data for all figures are provided as Supplementary Data 1. No restrictions apply to data availability.

Research involving human participants, their data, or biological material

Policy information about studies with human participants or human data. See also policy information about sex, gender (identity/presentation), and sexual orientation and race, ethnicity and racism.

Reporting on sex and gender	This study did not involve human participants, human data, or the use of human biological materials.
Reporting on race, ethnicity, or other socially relevant groupings	This study did not involve human participants, human data, or the use of human biological materials.
Population characteristics	This study did not involve human participants, human data, or the use of human biological materials.
Recruitment	This study did not involve human participants, human data, or the use of human biological materials.
Ethics oversight	This study did not involve human participants, human data, or the use of human biological materials.

Note that full information on the approval of the study protocol must also be provided in the manuscript.

Field-specific reporting

Please select the one below that is the best fit for your research. If you are not sure, read the appropriate sections before making your selection.

☒ Life sciences ☐ Behavioural & social sciences ☐ Ecological, evolutionary & environmental sciences

For a reference copy of the document with all sections, see nature.com/documents/nr-reporting-summary-flat.pdf

Life sciences study design

All studies must disclose on these points even when the disclosure is negative.

Sample size	For endophytic microbial community profiling, eight biologically independent replicates per condition were selected, considering the high variability and exploratory nature of immature microbiome techniques.For transcriptomic analysis, three biologically independent replicates were used per group, in accordance with standard practices in RNA-seq experiments due to the technical maturity and reproducibility of this approach.
Data exclusions	No data were excluded from the analyses.
Replication	All sequencing and biochemical measurements were based on independently collected biological samples (n = 3–8 per group). Analyses were conducted on all replicates without exclusions, and statistical trends remained consistent across samples.
Randomization	Moso bamboo shoot samples were collected randomly across biological replicates to avoid positional or temporal bias. Root, shoot bottom, and shoot top tissues were sampled from multiple individuals and randomized before sequencing and hormone measurements.
Blinding	Data analysts were blinded to sample identity during processing and statistical analysis to minimize potential bias.

Reporting for specific materials, systems and methods

We require information from authors about some types of materials, experimental systems and methods used in many studies. Here, indicate whether each material, system or method listed is relevant to your study. If you are not sure if a list item applies to your research, read the appropriate section before selecting a response.

Materials & experimental systems

n/a	Involved in the study
☒	☐ Antibodies
☒	☐ Eukaryotic cell lines
☒	☐ Palaeontology and archaeology
☒	☐ Animals and other organisms
☒	☐ Clinical data
☒	☐ Dual use research of concern
☐	☒ Plants

Methods

n/a	Involved in the study
☒	☐ ChIP-seq
☒	☐ Flow cytometry
☒	☐ MRI-based neuroimaging

Plants

Seed stocks

Moso bamboo (Phyllostachys edulis) shoots were collected from naturally growing stands in Wuxing District, Huzhou City, Zhejiang Province, China (latitude 30°48'N, longitude 119°59'E). Eight shoots of similar size and developmental stage were harvested on four different dates (January 4, February 24, March 1, and April 27, 2023), corresponding to distinct developmental stages. Each shoot was dissected into three compartments (shoot top, shoot bottom, and root), and samples were immediately frozen in liquid nitrogen for further analysis. No genetically modified organisms or commercial seed stocks were used.

Novel plant genotypes

No new genotypes were generated.

Authentication

Moso bamboo (Phyllostachys edulis) samples used in this study were collected from naturally growing populations in Wuxing District, Huzhou City, Zhejiang Province, China. The identity of P. edulis was confirmed based on morphological characteristics and local botanical records. No further molecular authentication was performed, as the species is well-characterized and distinguishable.
